# Neutrophil-related genes predict prognosis and response to immune checkpoint inhibitors in bladder cancer

**DOI:** 10.3389/fphar.2022.1013672

**Published:** 2022-10-21

**Authors:** Rui Yang, Wengang Zhang, Xiaoling Shang, Hang Chen, Xin Mu, Yuqing Zhang, Qi Zheng, Xiuwen Wang, Yanguo Liu

**Affiliations:** ^1^ Department of Medical Oncology, Qilu Hospital, Cheeloo College of Medicine, Shandong University, 107 Wenhuaxi Road, Jinan, Shandong, China; ^2^ School of Basic Medical Sciences, Shandong First Medical University, Jinan, China; ^3^ Department of Medical Imaging Center, Third People’s Hospital of Jinan, Jinan, China

**Keywords:** neutrophil, FCGRT, bladder cancer, immunotherapy, tumor microenvironment

## Abstract

Neutrophils play a key role in the occurrence and development of cancer. However, the relationship between neutrophils and cancer prognosis remains unclear due to their great plasticity and diversity. To explore the effects of neutrophils on the clinical outcome of bladder cancer, we acquired and analyzed gene expression data and clinical information of bladder cancer patients from IMvigor210 cohort and The Cancer Genome Atlas dataset (TCGA) database. We established a neutrophil-based prognostic model incorporating five neutrophil-related genes (EMR3, VNN1, FCGRT, HIST1H2BC, and MX1) and the predictive value of the model was validated in both an internal and an external validation cohort. Multivariate Cox regression analysis further proved that the model remained an independent prognostic factor for overall survival and a nomogram was constructed for clinical practice. Additionally, FCGRT was identified as the key neutrophil-related gene linked to an adverse prognosis of bladder cancer. Up-regulation of FCGRT indicated activated cancer metabolism, immunosuppressive tumor environment, and dysregulated functional status of immune cells. FCGRT overexpression was also correlated with decreased expression of PD-L1 and low levels of tumor mutation burden (TMB). FCGRT predicted a poor response to immunotherapy and had a close correlation with chemotherapy sensitivity. Taken together, a novel prognostic model was developed based on the expression level of neutrophil-related genes. FCGRT served as a promising candidate biomarker for anti-cancer drug response, which may contribute to individualized prognostic prediction and may contribute to clinical decision-making.

## 1 Introduction

Bladder cancer is one of the most common cancers globally, with an estimated 573278 new cases and 212,536 cancer deaths worldwide reported in 2020 (Sung et al., 2021). Based on clinical staging, bladder cancer can be divided into non-muscular invasive bladder cancer (NMIBC), muscular invasive bladder cancer (MIBC), and metastatic bladder cancer, each with different molecular drivers and specific treatment strategies (Sung et al., 2021). For decades, few advances have been made in bladder cancer therapeutics until the advent and application of immune checkpoint inhibitors (ICIs), which expand limited treatment options and show great promise, especially in patients with locally advanced or metastatic bladder cancer who develop intolerance or resistance to conventional first-line therapy (Wu et al., 2021). However, the improved outcomes brought about by ICIs are limited to selected patients and the prognosis remains poor. The 5-year overall survival rate for NMIBC is nearly 90%, compared with 60%–70% in MIBC and only 5%–30% in metastatic bladder cancer (Patel et al., 2020). Thus, effective immunotherapy indicators and prognostic biomarkers must be explored to identify bladder cancer patients who will benefit from ICI therapies and to facilitate risk stratification.

Neutrophils are key mediators of chronic inflammation and innate inflammatory response, as well as important regulators of cancer development (Hedrick and Malanchi, 2022). Instead of terminally differentiated cell populations, tumor-associated neutrophils are susceptible to environmental factors and exhibit great plasticity and diversity, which facilitate tumor growth or interfere with it (Jaillon et al., 2020). Accumulating evidence has suggested that neutrophils affect cancer prognosis and response to different modalities of anticancer treatment, ranging from chemotherapy to immunotherapy. Tumor-associated neutrophil infiltration, peripheral blood neutrophil-to-lymphocyte ratio, and neutrophil-based transcriptional signatures have generally been reported to correlate with poor clinical outcome and low drug sensitivity in many malignant diseases (Templeton et al., 2014; Gentles et al., 2015; Shaul and Fridlender, 2019). However, the prognostic role of neutrophils in bladder cancer remains unknown, nor is the predictive value of neutrophils in specific therapeutic regimens.

To investigate the effects of neutrophils on the prognosis of bladder cancer, we analyzed the gene expression profiles and clinical information of patients in the IMvigor210 cohort. A prognostic model based on neutrophil-related genes was established and validated. The relationships between neutrophil-related genes and the tumor microenvironment phenotype and drug sensitivity were further examined to identify potential immunotherapy response indicators and novel therapeutic targets.

## 2 Materials and methods

### 2.1 Data source and processing

IMvigor210 is an open-label, multicenter, single-arm Phase II clinical study evaluating the efficacy and safety of atezolizumab in patients with locally advanced or metastatic urothelial carcinoma who are not eligible for cisplatin-based chemotherapy. We downloaded clinical information and transcriptome data of 298 metastatic bladder cancer patients from http://research-pub.gene.com/IMvigor210CoreBiologies for our analysis. Overall, 298 patients were randomly divided into two data sets in a 7:3 ratio by the R package “Caret”: training set (*n* = 179) and internal validation set (*n* = 119). Data of 395 bladder cancer patients from The Cancer Genome Atlas dataset (TCGA) was downloaded as an external validation set.

### 2.2 Source of neutrophil-related gene

Neutrophil-related genes were retrieved from published studies, which can be classified into four gene sets: genes involved in neutrophil function, specificity, polarization, and infiltration, respectively. For genes involved in neutrophil function, 52 genes were extracted from the observation by Rincón et al. (Rincón et al., 2018). They were vital for the function of cell surface receptors, reactive oxygen species, guanine nucleotide exchange factors, and adhesion receptors in neutrophils. For genes involved in neutrophil specificity, 30 genes specifically expressed in neutrophils were obtained from research defining the immune landscape of human colon cancer (Bindea et al., 2013). For genes involved in neutrophil polarization, a continuum of neutrophil states (human N1–5) was identified in human lung cancer by single-cell transcriptomics and 30 genes enriched between the five neutrophil subsets were identified as neutrophil phenotype genes (Zilionis et al., 2019). Finally, 60 genes associated with neutrophil infiltration were obtained from the LM22, a leukocyte gene signature matrix applied in the CIBERSORT algorithm, which was widely used for the estimation of immune cell abundance from tissue expression profiles (Newman et al., 2015). After removing the duplicated genes in the four gene sets, a total of 172 genes were identified and they were defined as neutrophil-related genes for subsequent analyses.

### 2.3 Construction and validation of the risk model

Univariate COX regression was conducted in bladder cancer patients using the R packages “survival” and “SurvMiner” to select prognostic neutrophil-related genes, which could be used as candidates for construction of a prognostic model. The forest map of prognostic neutrophil-related genes was constructed using the R package “forestPlot.” The prognostic genes were then identified by Least Absolute Shrinkage and Selection Operator (LASSO) regression analysis *via* the R packages “glmnet” and a 5-gene risk model was established. The function “predict” in the R package “glmnet” was used to calculate the risk score of individual patients. Patients were divided into high- and low-risk groups according to the median risk scoreThe prognostic value of the 5-gene risk model was verified using the Kaplan-Meier curve and log-rank method *via* the R packages “survival” and “survminer”, and the sensitivity and specificity of the model were evaluated by the receiver operating characteristic (ROC) curve analysis *via* the R package “survivalROC”.

### 2.4 Nomogram construction

To determine the probability of survival predicted by the risk model and to facilitate its clinical use, a nomogram was drawn using the R package “rms.”

### 2.5 Survival analysis

Kaplan–Meier plots were drawn to elucidate the effect of each prognostic gene on overall survival (OS) of bladder cancer patients. The Log-rank test was used to evaluate their relationships.

### 2.6 Analysis of differentially expressed genes

The R package “limma” was used to select differentially expressed genes (DEGs). DEGs meeting the following criteria were considered significant: adjusted *p*-values <0.05 and |log2 fold change (FC)|≥0.5.

### 2.7 Pathway enrichment analysis

Gene Ontology (GO) and Kyoto Gene Genome Encyclopedia (KEGG) pathway analyses were performed *via* R package ‘clusterprofiler’, with a cutoff value of FDR <0.05. To study the differences in biological processes between the high and low expression groups of FCGRT, we applied gene set variation analysis (GSVA) with the R package “GSVA.”

### 2.8 Immune cell infiltration

To assess the composition of immune cells in the tumor microenvironment, we used the ESTIMATE algorithm to evaluate the immune score, tumor purity, and stromal score of tumor samples. The relative abundance of 24 tumor-infiltrating immune cells was obtained by single sample gene set enrichment analysis (ssGSEA) through the R package “GSVA.” We then obtained gene sets of exhausted CD8+T cells, naive NKT cells, and nonactivated NK cells from the Molecular Signatures Database (MSigDB) and calculated their enrichment using the GSEA algorithm.

## 3 Results

### 3.1 Establishment of the neutrophil-based prognostic model

**FIGURE 1:**
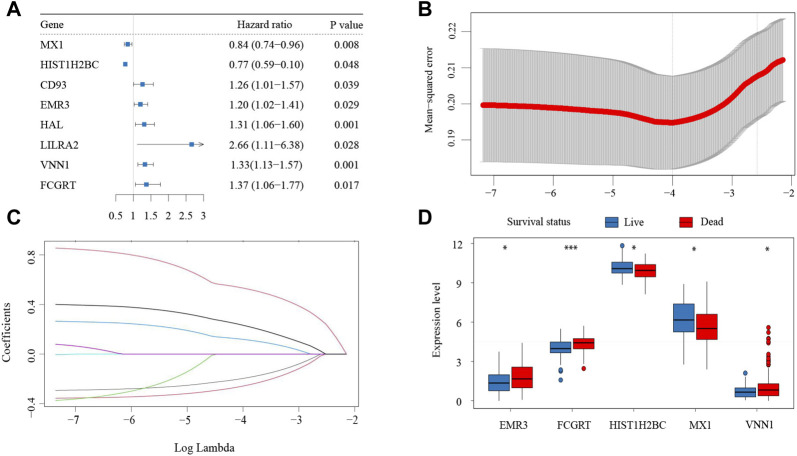
Establishment of the neutrophil-based prognostic model **(A)** Forest plot of eight neutrophil-related genes associated with survival. **(B)** Cross-validation for parameter selection **(C)** LASSO coefficient profile plot against the log lambda sequence. **(D)** Expression of the five prognostic genes in patients with different clinical outcomes. LASSO, least absolute shrinkage and selection operator.

To determine the correlation between neutrophil-related genes and overall survival (OS) of bladder cancer patients, a univariate Cox regression analysis was performed in the training cohort of the IMvigor210 dataset. Consequently, eight neutrophil-related genes were found to be associated with prognosis, including six genes detrimental to OS (CD93, EMR3, HAL, LILRA2, VNN1, FCGRT) and two genes favorable to OS (MX1, HIST1H2BC) (Figure 1A). These prognostic genes were then subjected to LASSO regression analysis to construct a neutrophil-based prognostic model. After calculating the active coefficients, the prognostic model achieved the best fit when λ points for -4 and 5 key prognostic genes (EMR3, VNN1, FCGRT, HIST1H2BC, and MX1) were incorporated (Figures 1B–D). The individual-level risk score determined by the prognostic model was used for risk stratification and prognosis prediction.

### 3.2 Validation of the neutrophil-based prognostic model

**FIGURE 2:**
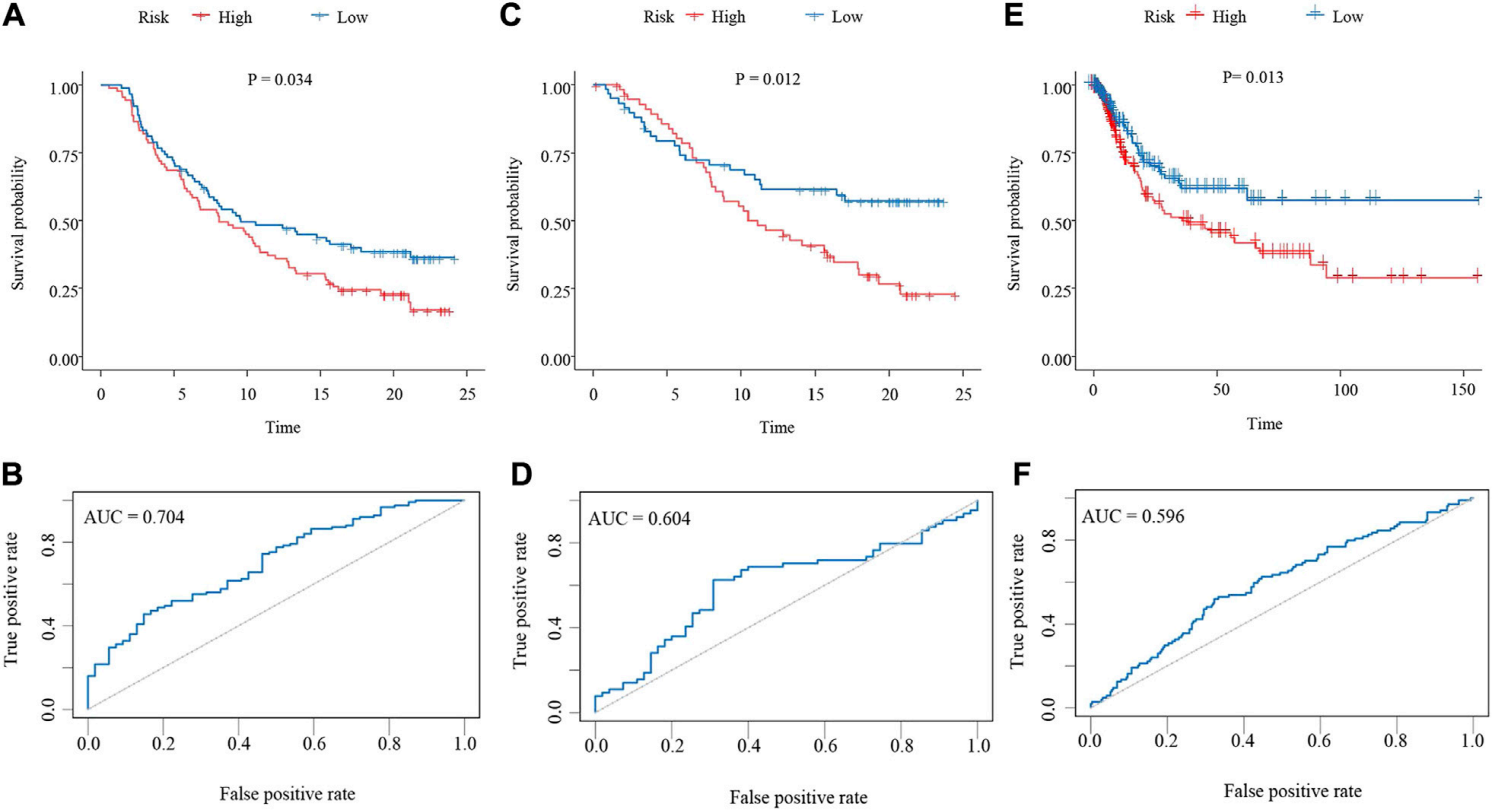
Validation of the neutrophil-based prognostic model **(A,C,E)** Kaplan–Meier survival curves of bladder cancer patients from the high-risk group and the low-risk group. **(A)**, training dataset (*p* = 0.034, HR = 1.309, 95%CI: 1.019–1.682). **(C)**, internal validation dataset (*p* = 0.012, HR = 1.578, 95%CI: 1.101–2.262). **(E)**, external validation dataset (*p* = 0.013, HR = 1.425, 95%CI: 1.076–1.889). **(B,D,F)** ROC curves evaluating the sensitivity and specificity of the neutrophil-based prognostic model. **(B)**, training dataset. **(D)**, internal validation dataset. **(F)**, external validation dataset. ROC, receiver operating characteristic.

The risk score for each patient in the training cohort was calculated using the neutrophil-based prognostic model and the median risk score of 0.69 was identified as the cutoff point. According to the cut-off point, bladder cancer patients in the training cohort were divided into high-risk and low-risk groups. Survival analysis showed that patients in the high-risk group had significantly shorter OS than patients in the low-risk group (*p* = 0.034, HR = 1.309, 95%CI: 1.019–1.682) (Figure 2A). Then a ROC curve was drawn to assess the predictive performance of the prognostic model. As shown in Figure 2B, the area under the curve (AUC) was 0.704. The analysis of the internal validation cohort from the IMvigor210 dataset corroborated these findings. Patients with a high-risk score had poorer clinical outcomes (*p* = 0.012, HR = 1.578, 95%CI: 1.101–2.262) and the AUC was 0.604 (Figures 2C,D). Furthermore, data from the TCGA dataset were downloaded as an external validation cohort. Consistent with the previous conclusions, the results showed that a high-risk score was closely related to an inferior OS (*p* = 0.013, HR = 1.425, 95%CI: 1.076–1.889) (Figures 2E,F).

### 3.3 Construction of predictive nomogram

**FIGURE 3:**
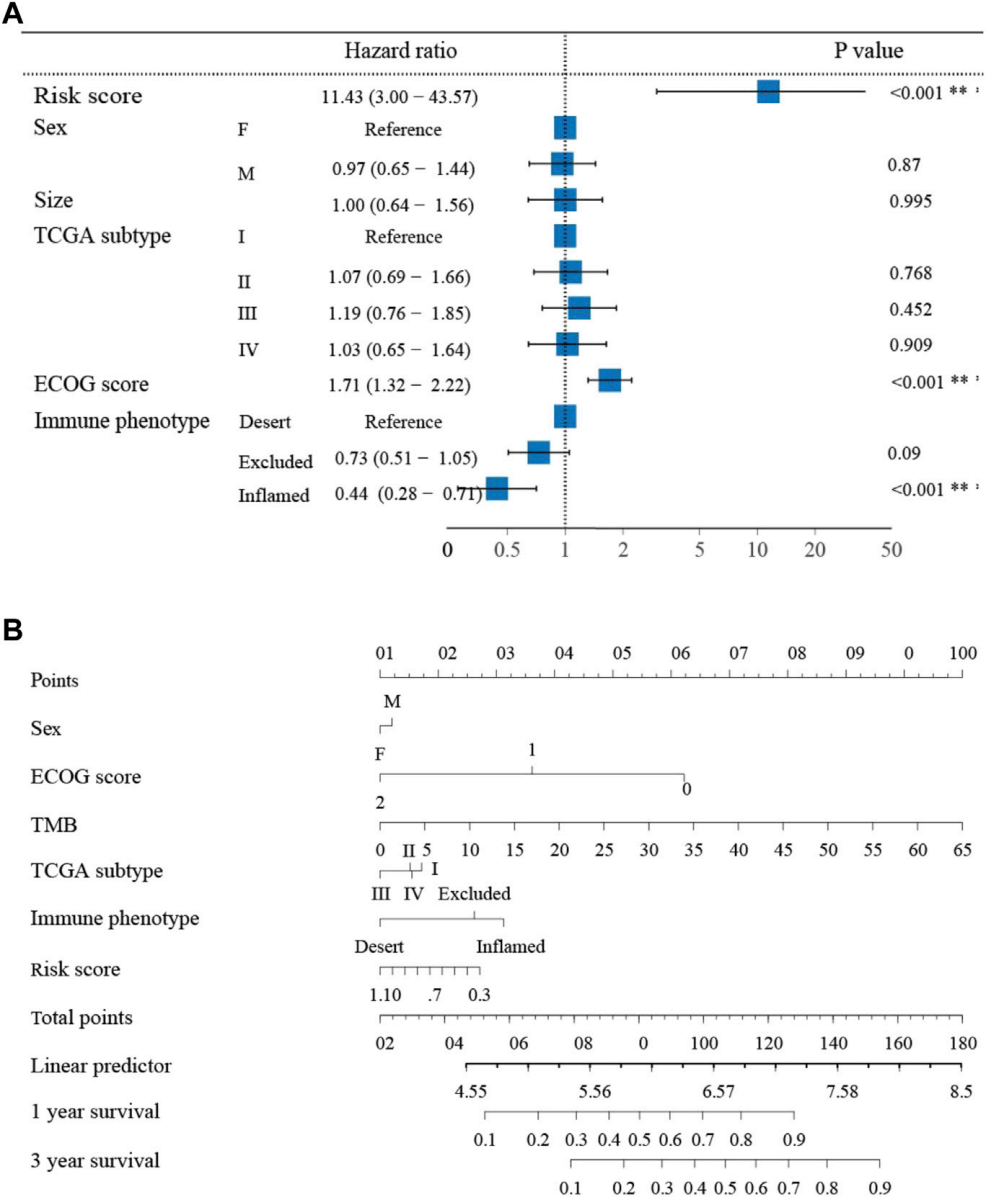
The nomogram to predict 1- and 3-year overall survival of bladder cancer patients **(A)** Multivariate Cox regression analysis revealing clinical characteristics related to prognosis. **(B)** The nomogram predicting overall survival of bladder cancer patients.

To further demonstrate the prognostic value of the neutrophil-based prognostic model, a multivariate Cox regression analysis was performed. The risk score was confirmed to be an independent prognostic factor for OS (HR: 11.43, 95% CI: 3.00–43.57, *p* < 0.001) (Figure 3A). Furthermore, the Eastern Cooperative Oncology Group (ECOG) score and immune phenotypes were also independent indicators of prognosis. Subsequently, for the convenience in clinical application, we established a nomogram to predict 1- and 3-year OS in bladder cancer patients. According to the results of the multivariate analysis, the risk score, the ECOG score and immune phenotypes were included in the nomogram. The total point scores for all parameters were matched with the survival time scales (Figure 3B).

### 3.4 Correlation of neutrophil-related genes with prognosis in bladder cancer

**FIGURE 4:**
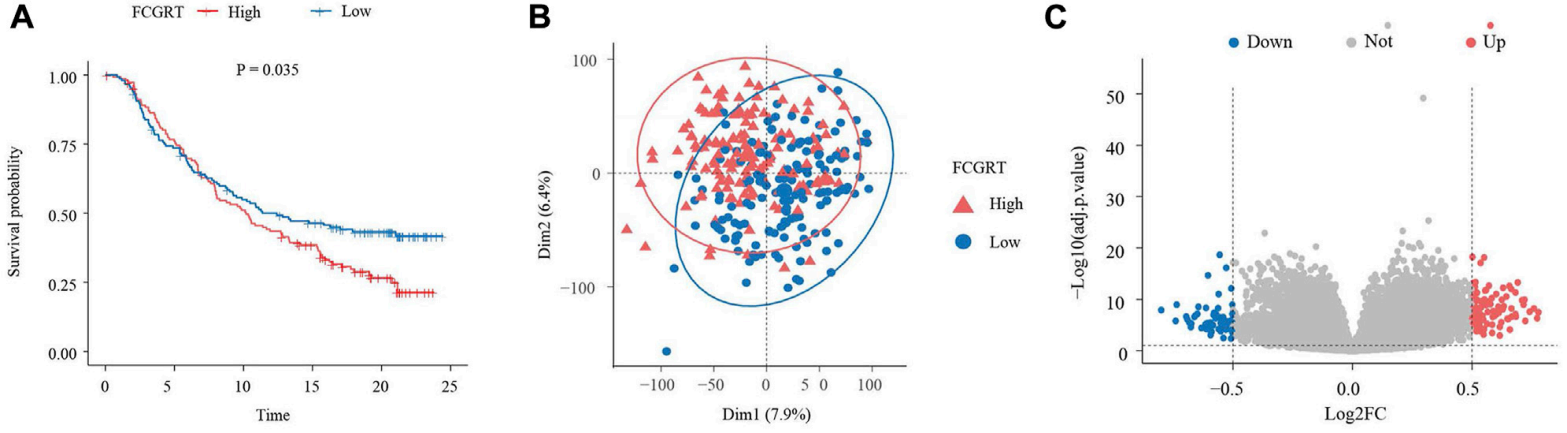
Identification of DEGs in FCGRT high and FCGRT low group **(A)** Kaplan–Meier survival curve of overall survival based on FCGRT expression (*p* = 0.035, HR = 1.245, 95%CI: 1.015–1.526) **(B)** Comparison of gene expression profiles of the FCGRT high and FCGRT low group. **(C)** The Volcano plot of DEGs. DEGs, differentially expressed genes.

With the aim of exploring the function of five neutrophil-related genes incorporated into the prognostic model, a survival analysis was performed. The Kaplan–Meier curves revealed that all five genes played a role in the prognosis of bladder cancer, while FCGRT had the strongest predictive power for survival (*p* = 0.035, HR = 1.245, 95%CI: 1.015–1.526) (Figure 4A; Supplementary Figure S1). To gain insight into the involvement of FCGRT in bladder cancer development, patients in the IMvigor210 cohort were divided into two groups based on the mean expression of FCGRT. The principal component analysis showed that the gene expression profiles were significantly different between the FCGRT high and FCGRT low groups (Figure 4B). The “limma” package was then used in the R environment to identify DEGs between the two groups. A total of 159 DEGs were selected with a cutoff *p*-value of 0.05 and |fold-change| >1.5 (Figure 4C).

### 3.5 Relationship between FCGRT expression and cancer metabolism and immunity

**FIGURE 5:**
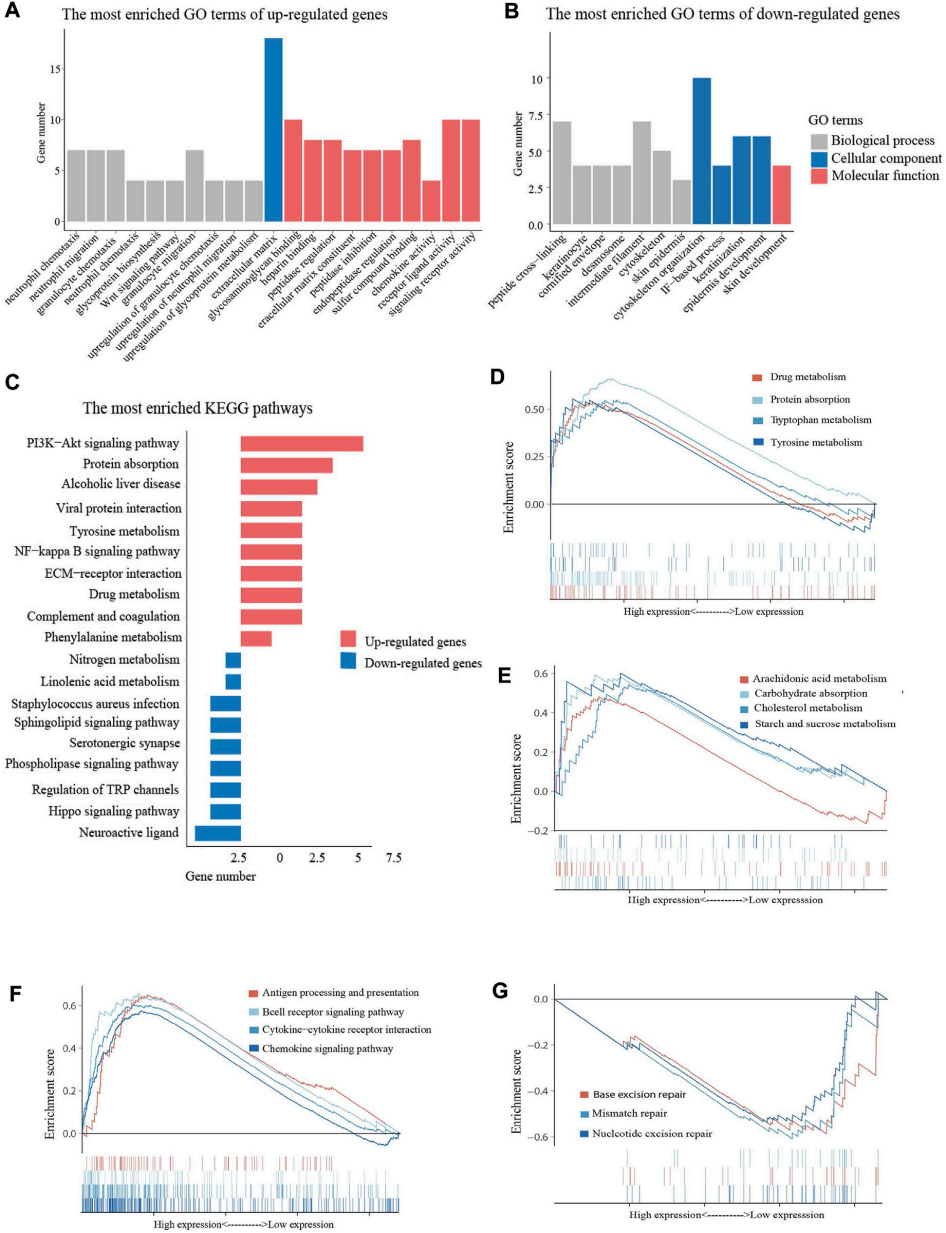
Enrichment analysis of DEGs **(A)** The most enriched GO terms of up-regulated DEGs. **(B)** The most enriched GO terms of down-regulated DEGs **(C)** The bar graph of the most enriched KEGG pathways. **(D,E)** Metabolism related pathways in GSEA enrichment analysis **(F)** Immunity related pathways in GSEA enrichment analysis. **(F)** DDR related pathways in GSEA enrichment analysis. DEGs, differentially expressed genes. GO, Gene Ontology. KEGG, Kyoto Encyclopedia of Genes and Genomes. GSEA, gene set enrichment analysis. DDR, DNA damage and repair.

Functional enrichment analysis was performed using the GO and KEGG databases to elucidate the biological functions of DEGs. GO enrichment analysis revealed that upregulated DEGs were mainly enriched in neutrophil chemotaxis and neutrophil migration, while downregulated genes were enriched in cytoskeleton organization and skin development (Figures 5A,B). KEGG enrichment analysis demonstrated that both upregulated and downregulated DEGs were involved in metabolism related pathways, such as protein digestion and absorption, tyrosine metabolism, and linolenic acid metabolism (Figure 5C). The GSEA analysis shared similar conclusions with the KEGG analysis. High expression of FCGRT was closely correlated with gene signatures that feature metabolic processe, including drug metabolism and nutrient metabolism (Figures 5D,E). Furthermore, the GSEA analysis showed that genes upregulated in the FCGRT high group were predominantly enriched in the immune process, such as antigen processing and presentation, the cytokine and chemokine signaling pathway, and B cell receptor signaling pathway (Figure 5F). The results allowed to reasonably infer that FCGRT was closely associated with antitumor immunity and remodeling of the tumor microenvironment. In addition, three mismatch repair pathways were suppressed in the FCGRT high group, suggesting a correlation between FCGRT and the DNA damage and repair process, which requires further exploration (Figure 5G).

### 3.6 FCGRT-shaped immunosuppressive context

**FIGURE 6:**
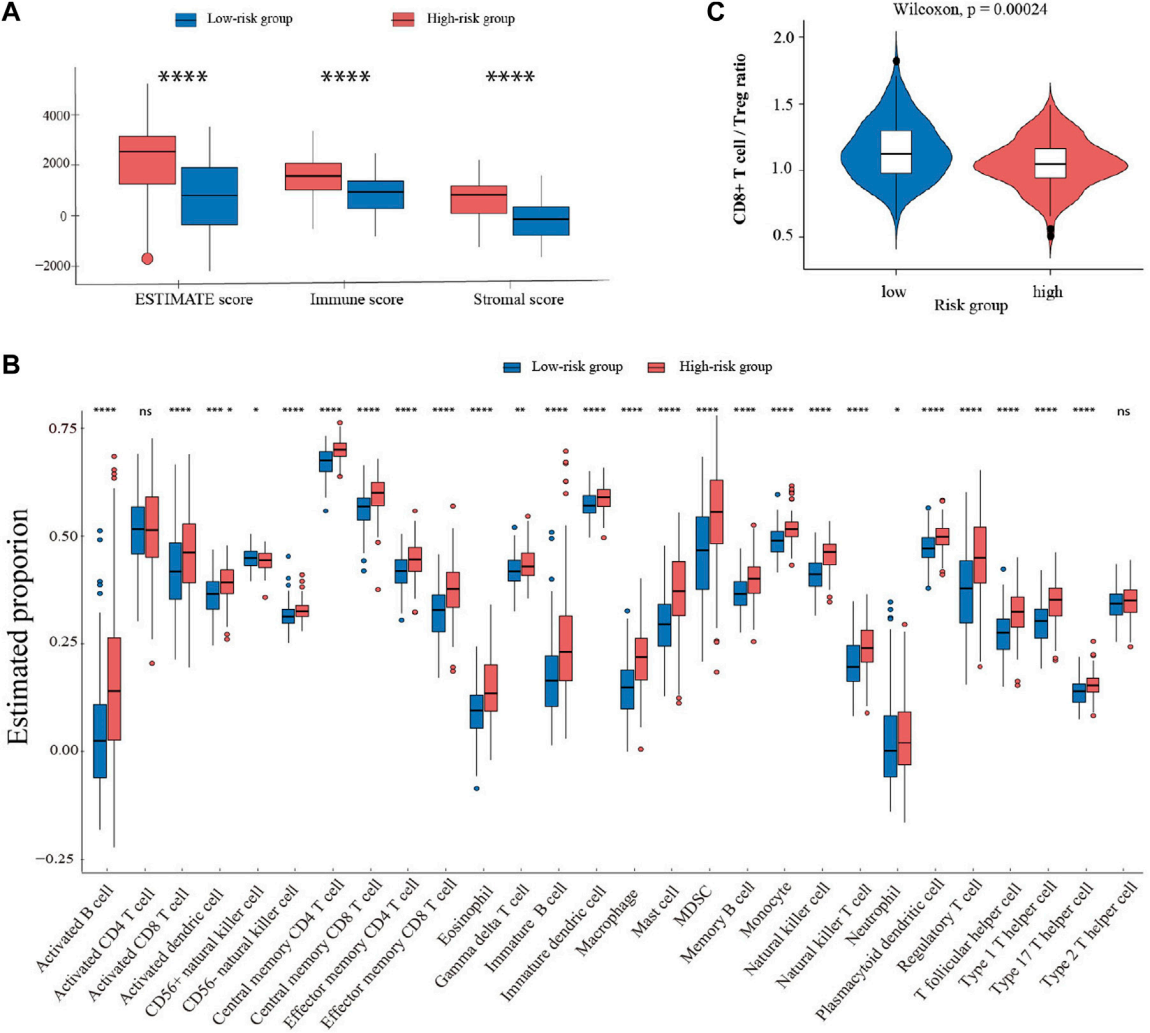
Comparison of immune profiles in two groups with different FCGRT expression **(A)** Boxplot of the ESTIMATE score. **(B)** The abundance of each immune cell type in the two groups. **(C)** The ratio of CD8+T/Treg cells in the FCGRT high group.

**FIGURE 7:**
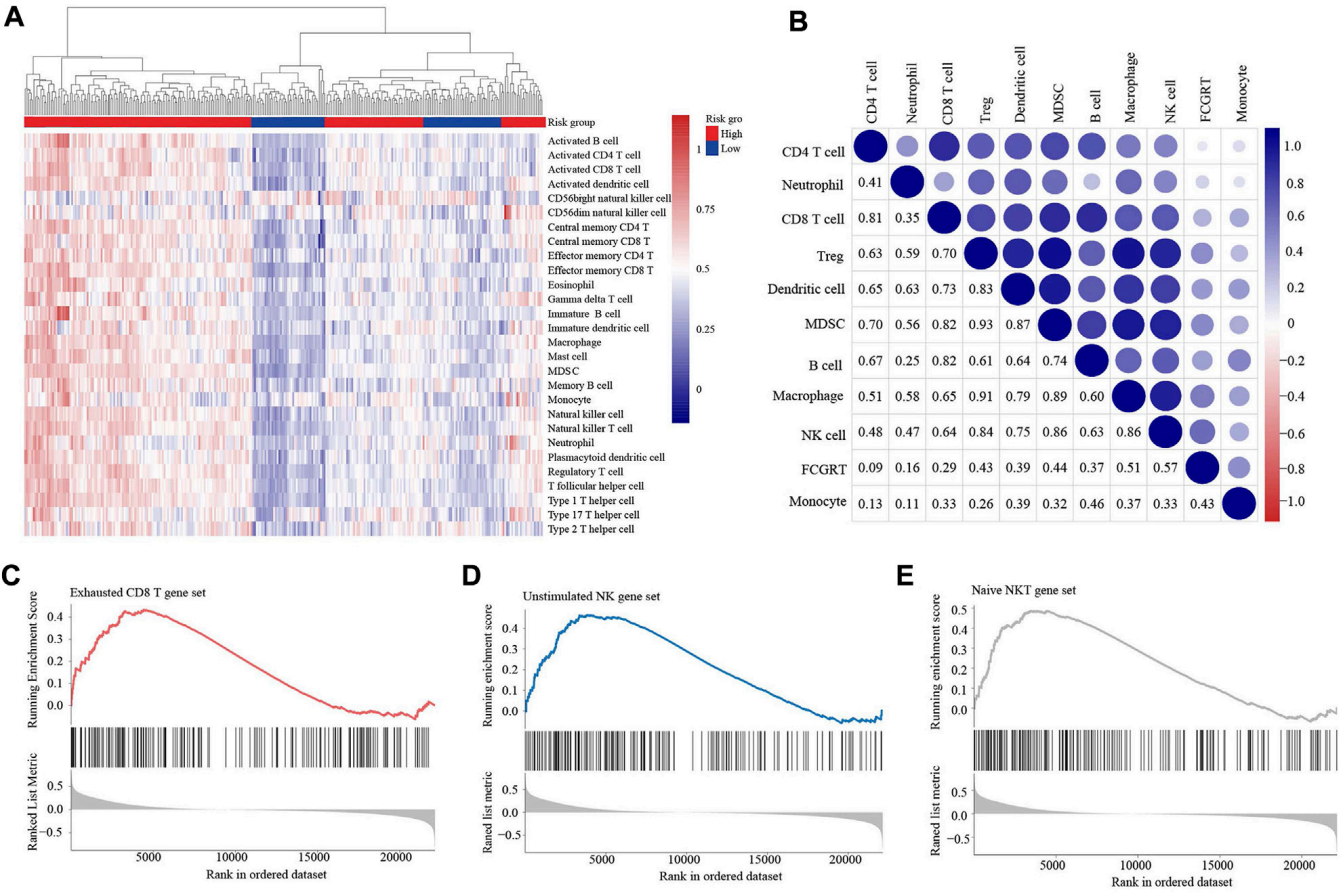
FCGRT-shaped immunosuppressive context **(A)** Heatmap of the infiltration profile of 28 immune cells in the two groups. **(B)** Correlation between FCGRT expression and immune cell infiltration. The *p*-value of the correlation is indicated in box. **(C–E)** The enriched gene sets in the FCGRT high group.

To explore the influence of FCGRT on the immune context of bladder cancer, ESTIMATE analysis was performed to evaluate the level of immune infiltration in tumor tissue. The results showed that the FCGRT high group had higher stromal and immune scores compared to the FCGRT low group (Figure 6A). ssGSEA analysis was then applied to calculate the proportion of each type of immune cells within tumor microenvironment (TME). The proportion of all tumor-infiltrating lymphocytes was significantly elevated in the FCGRT high group, except for activated CD4^+^ T cells and type 2 T helper cells (Figure 6B). However, the ratio of CD8^+^ T cells to Treg cells was decreased, indicating an immunosuppressive environment (Figure 6C). Then unsupervised group analysis revealed that the immune profiles of FCGRT high group and FCGRT low group were significantly different (Figure 7A). Further correlation analysis indicated close ties between FCGRT expression and immune cell infiltration, among which NK cells, macrophages and MDSCs exhibited the strongest association with FCGRT level (Figure 7B).

We also investigated the relationship between FCGRT expression and the functional status of immune cells. GSEA analysis showed that the exhausted CD8^+^ T cell gene set and the unstimulated NK cell gene set were significantly enriched in FCGRT high group (Figures 7C,D), implying that FCGRT could potentially orchestrate the dysfunction of CD8^+^ T cell and NK cell. Furthermore, the NKT cell, another important antitumor immune cell, was found to display a naïve phenotype in FCGRT high group (Figure 7E). Taken together, our results show that FCGRT influenced the tumor immune microenvironment through various mechanisms and shaped an immunosuppressive context in general.

### 3.7 Predictive value of FCGRT in responsiveness to anti-tumor drugs

**FIGURE 8:**
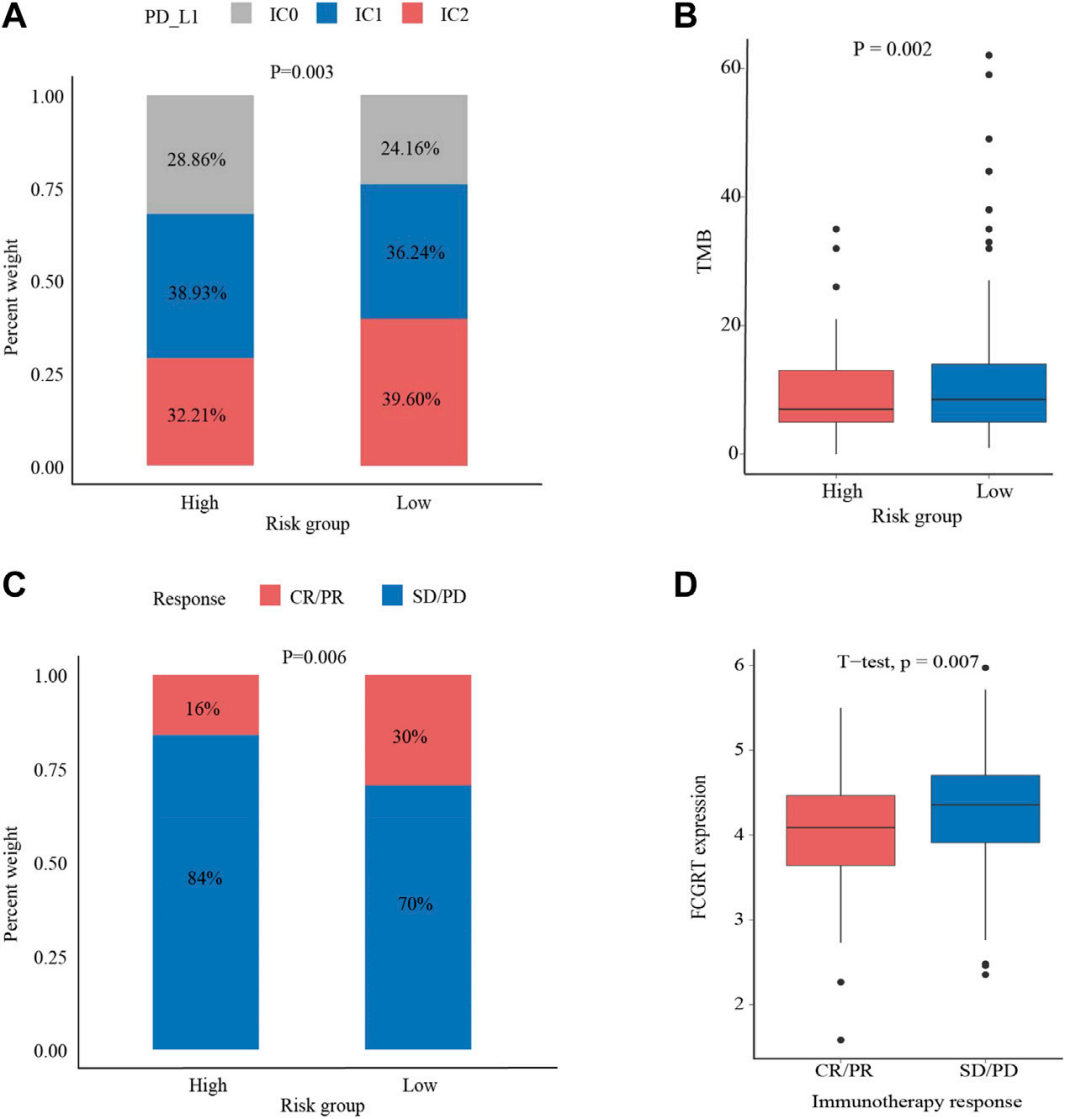
The correlation between FCGRT expression and response to immunotherapy **(A,B)** The correlation between FCGRT expression and PD-L1 and TMB level (*p* = 0.03, *p* = 0.002) **(C,D)** The correlation between FCGRT expression and immunotherapy sensitivity (*p* = 0.06, *p* = 0.007).

**FIGURE 9:**
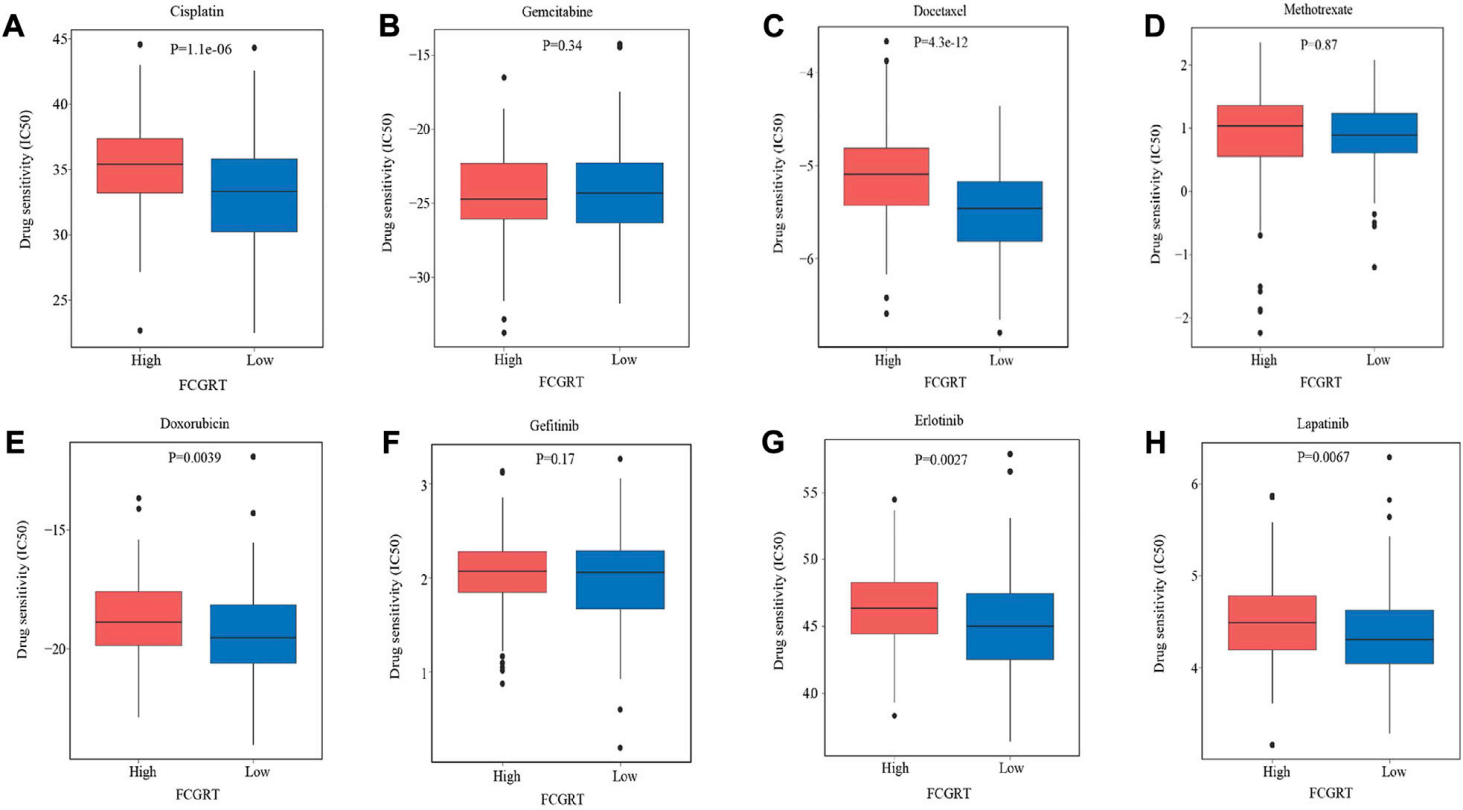
The correlation between FCGRT expression and response to chemotherapy and targeted therapy. **(A–H)** Estimated IC50 values of chemotherapy and targeted therapy drugs in FCGRT high and FCGRT low groups.

Given the immunophenotype discrepancy between the FCGRT high and FCGRT low group, it was hypothesized that FCGRT hold promise in predicting the immunotherapy response. We explored the correlation between FCGRT expression and recognized immunotherapy markers, including PD-L1 and the TMB. As shown in Figures 8A,B, patients with high FCGRT showed reduced PD-L1 expression and decreased TMB level (*p* = 0.003, *p* = 0.002). The overall response rate of FCGRT high group was only 16%, which was much lower than 30% of the FCGRT low group (*p* = 0.006) (Figure 8C). Furthermore, patients who responded to ICIs exhibited decreased FCGRT level (*p* = 0.007) (Figure 8D). These results suggested that FCGRT could serve as a potential biomarker of poor immunotherapy efficacy in bladder cancer. We also analyzed the correlation between FCGRT expression and sensitivity to chemotherapy and targeted therapy. The results showed that the estimated IC50 values of cisplatin, docetaxel, doxorubicin, erlotinib and lapatinib were significantly higher in the FCGRT high group, while the estimated IC50 values of gemcitabine, methotrexate, and gefitinib did not show statistical differences between the two groups (Figures 9A–H).

## 4 Discussion

Bladder cancer is the most common malignancy of the urinary system (Bellmunt et al., 2022). Despite advances in targeted therapy and immune regimen, the prognosis of bladder cancer remains unfavorable. Therefore, effective prognostic and therapeutic markers are needed to optimize disease management. The current risk stratification of bladder cancer is based on clinicopathological features, such as clinical stages and tumor grades, none of which exhibits high specificity and sensitivity (Soria et al., 2019). Furthermore, these conventional prediction tools have focused primarily on the intrinsic characteristics of cancer, ignoring the contribution of TME to cancer genesis and development (Kluth et al., 2015). Tumor-associated neutrophils are critical components of TME and its involvement in cancer development has become more and more prominent in recent years. Neutrophils play an important role in the initiation, development and progression of bladder cancer (Thompson et al., 2015). They can directly or indirectly regulate bladder cancer development by boosting inflammation, promoting angiogenesis, and mediating immunosuppression. Neutrophils also play a role in predicting treatment responses and prognosis of bladder cancer. Neutrophils and neutrophil extracellular traps (NETs) have been reported to influence the efficacy of BCG immunotherapy and mediate resistance to radiation therapy (Liu et al., 2019; Shinde-Jadhav et al., 2021). High circulating neutrophil counts and elevated neutrophil-to-lymphocyte ratio (NLR) are associated with adverse clinical outcomes for patients with bladder cancer (Tang Du et al., 2017). Additionally, tumor-infiltrating neutrophils exhibit pro-carcinogenic effects and predict poor prognosis (Liu et al., 2018). To further elucidate the relationship between neutrophils and the prognosis of bladder cancer, we analyzed the transcriptomic data from the IMvigor210 cohort and constructed a prognostic model based on the expression profile of neutrophil-related genes. Moreover, FCGRT was found to participate in cancer metabolic reprogramming and mediate an immunosuppressive tumor microenvironment. Furthermore, we validated the significance of FCGRT in predicting responses to anti-cancer drugs in bladder cancer.

Advances in next-generation sequencing (NGS) technology allow access to genomic profiles of cancer. The application of NGS has identified multiple mutations and gene expression signatures that aid in precise cancer classification and prognosis prediction. Previous studies have shown that immune cell related genes were closely correlated with clinical outcomes of bladder cancer, such as myeloid cells and antitumor T cells (Eckstein et al., 2020; Wang et al., 2021). In the current study, we first investigated the role of neutrophil-related genes in the prognosis of bladder cancer. It was found that eight neutrophil-related genes, namely CD93, EMR3, HAL, LILRA2, VNN1, FCGRT, MX1, and HIST1H2BC, had a strong correlation with the prognosis of bladder cancer. Among them, CD93 and HIST1H2BC were specifically expressed in neutrophils and constituted the distinctive transcriptional profile of neutrophils, facilitating the identification of neutrophils within the tumor microenvironment (Bindea et al., 2013). VNNI, EMR3, LILRA2, and HAL all played significant roles in neutrophil infiltration (Newman et al., 2015). They were widely employed to quantify the number of infiltrating neutrophils in various tissues or malignancies. FCGRT was critical to neutrophil function (Patel and Bussel, 2020). The FCGRT gene-encoded neonatal Fc receptor for IgG (FcRn) participated in IgG-mediated antigen phagocytosis by neutrophils, hence enhancing immune defenses against pathogenic pathogens. MX1 was closely related to the phenotypic heterogeneity of neutrophils (Zilionis et al., 2019). According to single-cell transcriptomics, human neutrophils could be divided into five subsets (N1-N5), and MX1 was prominently enriched in the N2 neutrophil subtype and served as a marker gene for this specific neutrophil population.

Incorporated five genes (MX1, HIST1H2BC, EMR3, VNN1, and FCGRT), an effective prognostic model was constructed. MX1 is an interferon-induced GTPase localized in the cytoplasm. MX1 had been studied mainly for its antiviral properties until recent studies reported its biological functions in tumorigenesis (Calmon et al., 2009). *In vitro* experiments revealed that MX1 was able to inhibit cancer cell migration and reduce metastasis of prostate and melanoma cancer (Mushinski et al., 2009). HIST1H2BC is a member of the histone H2B family. Emerging evidence has shown that histone modification plays a key role in tumorigenicity, and mutations in histone H2B have been identified as important cancer drivers (Bajbouj et al., 2021; Markouli et al., 2021). However, few reports have described its correlation with cancer prognosis, which deserves further investigation. EMR3, an adherent G protein-coupled receptor, is highly expressed in neutrophils, monocytes, and macrophages (Matmati et al., 2007). EMR3 has been reported to mediate the aggressive phenotype of glioblastoma (Kane et al., 2010). The knockdown of EMR-3 reduced cell invasion capacity by more than three times. VNN1 encodes the Vnn1 protein, whose main function is to inhibit caspase and scavenge reactive oxygen species (Naquet et al., 2014). Vnn1 has been reported to play an important role in tumorigenesis. VNN1 deficient mice developed resistance to oxidative stress, thus inhibiting intestinal inflammation and reducing the incidence of colorectal cancer (Pouyet et al., 2010). FCGRT encodes the α-chain of the neonatal Fc receptor (FcRn), a transporter of immunoglobulin G (IgG) and albumin. FcRn maintains serum IgG and albumin levels in a pH-dependent manner. It also plays a vital role in antigen phagocytosis and presentation of immune complexes (Vidarsson et al., 2006; Patel and Bussel, 2020). Dysregulation of FcRn has been reported to exert effects on the prognosis of non-small cell lung cancer, colorectal cancer, and hepatocellular carcinoma (Dalloneau et al., 2016; Shi et al., 2016). The biological functions of FCGRT in bladder cancer have yet to be elucidated.

Metabolic reprogramming is a hallmark of cancer and an important contributor to cancer progression. The metabolic switch allows the uptake of deregulated nutrients and efficient energy supply to fuel cancer cell growth and division (Martínez-Reyes and Chandel, 2021). In our study, FCGRT was found to alter cancer metabolism by modulating metabolic signaling pathways in both malignant and non-malignant cells within TME. The metabolic changes in neutrophils were able to directly or indirectly influence the TME. For instance, Rice et al. (2018) found that the mitochondrial activity of neutrophils was enhanced in malignant diseases, leading to increased intracellular NADPH levels and ROS production. The overexpressed ROS hindered T cell activity and created an immunosuppressive environment. In our study, it was observed that FCGRT was correlated with the activation of ROS-related pathways, such as the HIF-1 signaling pathway and oxidative phosphorylation, which might be involved in the complex interaction between neutrophils and TME. Alteration of lipid metabolism in neutrophils also plays an important role in TME remolding. Li et al. found neutrophils at metastatic cancer sites had increased lipid storage, and these accumulated lipids within neutrophils can be transported to metastatic cancer cells *via* a micropinocytosis–lysosome pathway, which subsequently facilitated tumor proliferation and enhanced metastatic environment (Li et al., 2020). The upregulated lipid metabolism mediated by FCGRT might similarly contribute to the development of an immunosuppressive TME.

It is speculated that FCGRT regulates cancer metabolism through the engagement of FcRn with albumin transport (Cadena Castaneda et al., 2020). Albumin is the most abundant protein in the circulation and functions as an endogenous transporter for multiple small molecules such as fatty acids, amino acids, bilirubin, and drugs (Rabbani and Ahn, 2019). Overexpressed FcRn leads to increased albumin recycling, during which the nutrient cargo carried by albumin is released intracellularly and promotes the metabolism and proliferation of cancer cells. These findings were corroborated in human cancer epithelial cells (Larsen et al., 2020). The albumin recycling process was reported to be highly FcRn-dependent and FcRn knockout decreased the metabolic rate of malignant cells *in vitro*, as well as cancer growth *in vivo*. Furthermore, we found that genes related to drug metabolism were also predominantly enriched in FCGRT high group. The underlying mechanism is that FcRn-mediated transcellular recycling mechanism protects albumin-binding drugs from degradation by serum and tissue proteases. The protective role of FcRn extends the circulatory half-life of albumin-binding drugs, and improve their uptake and utilization by target tissues (Yu et al., 2022). New treatment perspectives have been suggested based on the mechanism. Albumin-based drug conjugates were engineered with different affinity for FcRn to fine tune pharmacokinetics, thus achieving an optimal therapeutic profile (Larsen et al., 2018).

Despite its participation in cancer metabolic reprogramming, FCGRT also plays a role in cancer immunity (Coffelt et al., 2016). In our study, FCGRT expression was significantly correlated with gene signatures featuring immune regulation, including the chemokine signaling pathway, antigen processing and presentation, and immune cell migration and function. The FcRn-mediated immunosurveillance has been in colorectal cancer (Baker et al., 2013). It was found that FcRn expression within dendritic cells induced IL-12 production and promoted the cross-priming of tumor antigens to CD8 + T cells, thus improving antitumor immunity and inhibiting tumor development. In addition to immune regulation, FcRn expression also affected tumor microenvironment landscapes. Tumors with high expression of FCGRT exhibited increased infiltration by a number of immune cell subtypes, compared to tumors with low expression of FCGRT. This phenomenon indicated the potential role of FCGRT in the identification of an immune-inflamed phenotype (Gerard et al., 2021). Consistent with our study, Castaneda et al. revealed that the proportion of tumor-infiltrating dendritic cells, CD8^+^ T cells, and NK cells was significantly reduced in FcRn-knockout mice compared to wild-type mice (Castaneda et al., 2018). Although the number of immune activating and immune suppressive cells was both elevated in FCGRT high group, the ratio of CD8^+^ T cells to Treg cells was decreased, indicating a suppressive TME in general. Furthermore, FCGRT could potentially orchestrate the dysfunction of antitumor immune cells. FCGRT was able to alter the cytotoxic activity of antitumor immune cells, with the enrichment of exhausted CD8^+^ T cell gene set, unstimulated NK gene set, and naive NKT gene set in FCGRT high group. However, our results were opposite to existing research indicating that FcRn promoted NK cell maturation and interferon γ production (Castaneda et al., 2018). More studies are needed to clarify the role of FCGRT in the development and activation of immune cells.

ICIs have revolutionized the treatment of bladder cancer and extended patient survival to an unprecedented extent. However, not all patients can benefit from immune therapy. We found that FCGRT could serve as a potential biomarker for immunotherapy. Bladder cancer patients with high expression of FCGRT had a lower response rate to ICIs. ICI therapies are designed to reinvigorate the effective antitumor immune response mediated by T cells. Therefore, the functionality of CD8^+^ T cells determines whether patients are likely to respond to ICI (Waldman et al., 2020). Overexpressed FCGRT altered T cell activation and priming. T cell dysfunction interferes with the development of a robust antitumor response after ICI treatment and weakens the efficacy of immunotherapy. Furthermore, FCGRT expression was found to correlate with a decrease in PD-L1 expression and a low TMB level, which collectively indicated an unfavorable response to immunotherapy (Shum et al., 2022). PD-L1 expression is the most intuitive predictive biomarker of ICI and has been widely used for patient selection in clinical practice. TMB reflects the level of cancer mutation and the ability to produce neoantigen by malignancies (Chan et al., 2019; Li et al., 2020). The lower the TMB, the less likely that T cells will recognize and eradicate tumor antigens. In addition to immunotherapy, FCGRT was also correlated with high sensitivity to chemotherapy and targeted agents. The alteration of the DNA damage and repair (DDR) pathways mediated by FCGRT may represent an underlying mechanism. Most chemotherapy drugs disrupt DNA integrity or interfere with DNA synthesis to inhibit cancer cell replication. Defects in the DDR pathways make it difficult to detect and repair DNA lesions and improve the therapeutic efficacies of chemotherapy (Goldstein and Kastan, 2015; Reisländer et al., 2020).

There are still some limitations in our study. First, further verification of our conclusions is limited by insufficient data. Clinical samples and prospective studies are warranted for further validation. Second, there is a close link between neutrophil-related genes and tumor microenvironment observed in our research. The underlying mechanism remains unclear and requires investigation in the future. Third, neutrophil-related genes used in our analysis should be more comprehensive and updated to ensure the authenticity of the research.

## 5 Conclusion

We established a prognostic model that contains five neutrophil-related genes in bladder cancer and proved its possible independent prognostic value. In addition, we found FCGRT-mediated cancer metabolic reprogramming and tumor microenvironment remodeling, which could be used as a novel biomarker for ICIs therapy. Future studies should be conducted to clarify the function of FCGRT in bladder cancer and verify the predictive capacity of the neutrophil-based model in clinical applications.

## Data Availability

The datasets presented in this study can be found in online repositories. The names of the repository/repositories and accession number(s) can be found in the article/Supplementary Material.

## Abbreviations

NMIBC, non-muscular invasive bladder cancer; MIBC, muscular invasive bladder cancer; ICIs, immune checkpoint inhibitors; TCGA, The Cancer Genome Atlas dataset; LASSO, Least Absolute Shrinkage and Selection Operator; ROC, the receiver operating characteristic; DEGs, differentially expressed genes; GO, Gene Ontology; KEGG, Kyoto Gene Genome Encyclopedia; GSVA, gene set variation analysis; ssGSEA, single sample gene set enrichment analysis; OS, overall survival; AUC, area under the curve; ECOG, Eastern Cooperative Oncology Group; TME, tumor microenvironment. NGS, next-generation sequencing.
