# Supplementary figures and images for: Neutrophil-related genes predict prognosis and response to immune checkpoint inhibitors in bladder cancer
Source: Front Pharmacol. 2022 Oct 21;13:1013672. doi: 10.3389/fphar.2022.1013672 (PMC9635818; doi:10.3389/fphar.2022.1013672)

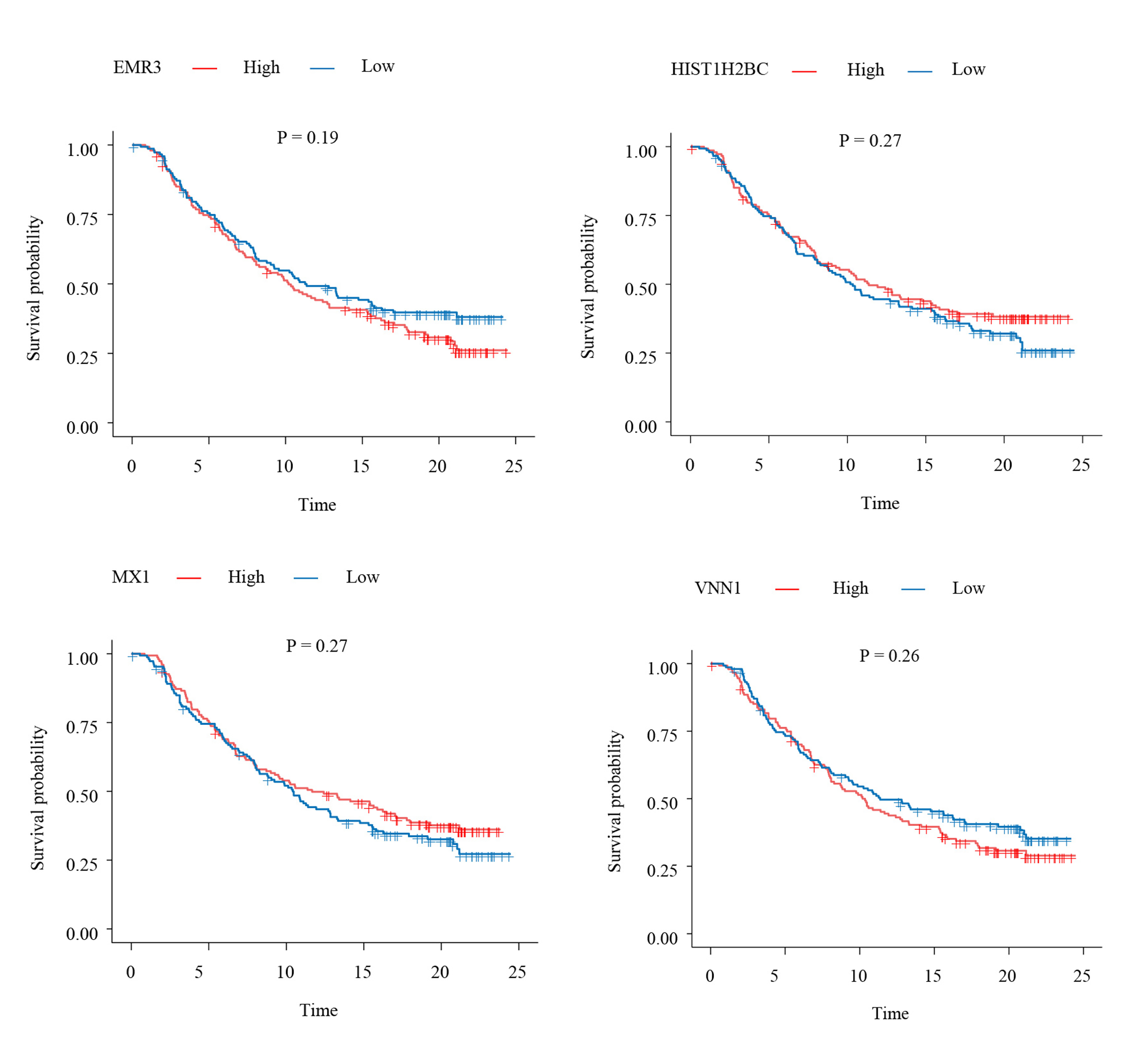

Supplement: Supplementary file 1 [file Image1.JPEG]
